# The SIB Swiss Institute of Bioinformatics’ resources: focus on curated databases

**DOI:** 10.1093/nar/gkv1310

**Published:** 2015-11-28

**Authors:** SIB Swiss Institute of Bioinformatics Members

**Affiliations:** SIB Swiss Institute of Bioinformatics, Quartier Sorge, Bâtiment Génopode, CH-1015 Lausanne, Switzerland; University of Basel, Klingelbergstrasse 50–70, CH-4056 Basel, Switzerland; Friedrich Miescher Institute for Biomedical Research (FMI), Maulbeerstrasse 66, CH-4058 Basel, Switzerland; Swiss Tropical and Public Health Institute, Socinstrasse 57, CH-4051 Basel, Switzerland; Institute of Oncology Research, Via Vincenzo Vela 6, CH-6500 Bellinzona, Switzerland; University of Bern, Baltzerstrasse 6, CH-3012 Bern, Switzerland; University of Fribourg, Chemin du Musée 10, CH-1700 Fribourg, Switzerland; University of Geneva, CMU, Rue Michel-Servet 1, CH-1211 Geneva 4, Switzerland; HES-SO, HEG Genève, route de Drize 7, CH-1227 Carouge, Switzerland; Ecole Polytechnique Fédérale de Lausanne (EPFL), CH-1015, Lausanne, Switzerland; Ludwig Institute for Cancer Research, CH-1015 Lausanne, Switzerland; University of Lausanne, CH-1015 Lausanne, Switzerland; Università della Svizzera Italiana, Via Giuseppe Buffi 13, CH-6900 Lugano, Switzerland; Agroscope, Schloss 1, P.O. Box, CH-8820 Wädenswil, Switzerland; Zurich University of Applied Sciences, Grüental, P.O. Box, CH-8820 Wädenswil, Switzerland; ETH Zurich, Universitätstrasse 6, CH-8006 Zurich & Mattenstrasse 26, CH-4058 Basel, Switzerland; University of Zurich, Winterthurerstrasse 190, CH-8057 Zurich, Switzerland

## Abstract

The SIB Swiss Institute of Bioinformatics (www.isb-sib.ch) provides world-class bioinformatics databases, software tools, services and training to the international life science community in academia and industry. These solutions allow life scientists to turn the exponentially growing amount of data into knowledge. Here, we provide an overview of SIB's resources and competence areas, with a strong focus on curated databases and SIB's most popular and widely used resources. In particular, SIB's Bioinformatics resource portal ExPASy features over 150 resources, including UniProtKB/Swiss-Prot, ENZYME, PROSITE, neXtProt, STRING, UniCarbKB, SugarBindDB, SwissRegulon, EPD, arrayMap, Bgee, SWISS-MODEL Repository, OMA, OrthoDB and other databases, which are briefly described in this article.

## INTRODUCTION

Like physics and astronomy, many life science disciplines are continuously producing more and more data. The interpretation of these data requires significant information technology resources, such as high performance computing (HPC), software solutions, the capacity to store data, transform them into knowledge, and make the accumulated knowledge both available and easily findable. Moreover, along with experimentation and theory, computational simulation has become a third pillar of science, allowing researchers to advance their understanding of complex systems *in silico*. From understanding the 3D structure of macromolecules to designing drugs and mapping molecular pathways, bioinformatics continues to be at the forefront of many fields of life science research. As the data deluge from genomics may well outgrow the amount of data produced in astronomy (1,2), the importance of bioinformatics is expected to increase even further in the coming years.

**Figure 1.**
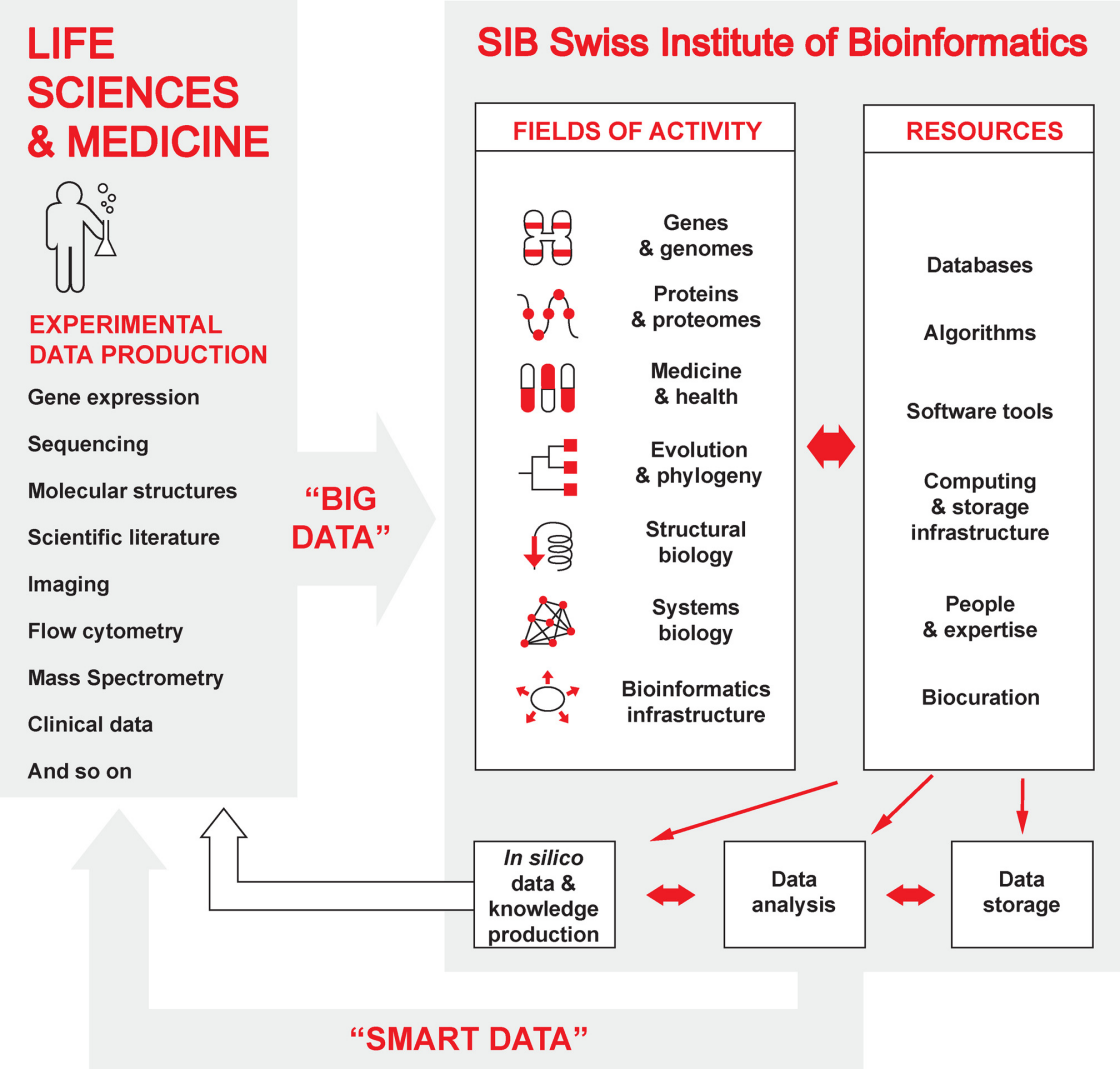
Overview of SIB's fields of activity and resources related to big data, in the life sciences and health.

The scientists at the SIB Swiss Institute of Bioinformatics contribute to this field with expertise in data management, storage, integration and analysis, thereby addressing the needs of the scientific community in both academia and industry (Figure 1). The institute not only leads but also coordinates bioinformatics in Switzerland, federating researchers and delivering training. What is more, its data science experts provide the national and international life science community with a state-of-the-art bioinformatics infrastructure, including resources, expertise and services. The several hundred internationally recognized databases and software tools that SIB develops and maintains, are part of this infrastructure. SIB is internationally renowned for resources such as UniProtKB/Swiss-Prot (3), STRING (4) and SWISS-MODEL (5,6). Its databases and knowledge bases give life scientists access to curated biological data and information. Its software tools allow the integration, analysis, visualization, interpretation and comparison of biological data as well as the means to model biological systems, and thus play an essential role in turning the data into knowledge.

The institute has an important high-quality biocuration activity, thus providing life scientists with accurate and comprehensive representations of biological knowledge. Through its national and international training activities, which are open to all scientists, SIB strives to instill and maintain a high level of bioinformatics knowledge in current and future generations. It also ensures the informed and efficient use of public bioinformatics resources.

SIB federates some 60 service and research groups spread across Switzerland, including almost 700 members who are also affiliated to the major Swiss Schools of Higher Education and research institutes. Such a high concentration of bioinformatics groups is unique in the world. The scientific scope of SIB's groups spans a large variety of life science domains such as genomics, proteomics, systems biology, structural biology, evolutionary biology and medicine as indicated in the ‘fields of activity’ in Figure 1. Here, we provide an overview of its current competence areas and corresponding scientific resources and services. All SIB groups are regularly invited to list their resources on ExPASy, SIB's bioinformatics resource portal www.expasy.org (7). Created in 1993, ExPASy was at the time the very first website available in the biomedical field. Following SIB's model, ExPASy federates over 150 SIB resources, and a few external resources for historical reasons. It supports interoperable queries, thus facilitating rapid and easy access to multiple databases. New resources are continuously added once published. While dedicated teams provide long-term support to the resources already available through ExPASy; and most resources provide some form of user support.

In 2014, on the occasion of SIB's 15th anniversary, we provided an overview of its core resources (8). In the present article, we focus on the added value of biocuration at SIB and on the SIB resources that provide this service. Additionally, we outline the main competence areas and directions SIB is working on.

## THE VALUE OF BIOCURATION

Most life science databases can be classified as either *primary databases* or *secondary databases* (also known as *knowledge bases*).

*Primary databases* are composed of original archival data submitted directly by data producers. They are characterized by very high data volumes and rates of growth, and minimal curation often done by the data producers themselves. Typical examples of primary databases are the European Nucleotide Archive (ENA) (9) and the Sequence Read Archive (SRA) (10) which store raw data from sequencing technologies, or the Gene Expression Omnibus database (GEO) (11), a data repository for high-throughput gene expression data. Primary databases also play an increasingly important role in the proteomics field, including solutions like PRIDE for tandem MS/MS data (12) and Passel for Selected Reaction Monitoring data (13).

*Secondary databases or knowledge bases* are derived from primary databases through either automated annotation or manual, expert curation processes. The UniProt Knowledgebase UniProtKB provides examples of both types of database; UniProtKB/TrEMBL contains protein sequences enriched with computational annotation, while UniProtKB/Swiss-Prot is curated by human experts. These biocurators summarize the findings from key articles in a form that can be easily queried as well as linked to other data types by using shared standards such as resource identifiers and ontologies (14). The quality and utility of the information provided is far superior to that which is achieved by using computational means (15). Biocuration thus requires a combination of human intelligence, well-designed software tools, and advanced computational methods for literature identification and triage. Such a combination helps human curators to keep pace with the ever growing body of biological knowledge (approximated by the rapid growth of published literature).

Expert biocuration (16,17) is a crucial part of SIB's mission to provide world-class resources and infrastructure for life scientists. SIB supports a large community of expert biocurators, bioinformaticians and software developers who, together, have sustained the development of renowned knowledge resources like Swiss-Prot, which celebrates its 30th anniversary in 2016.

In the remainder of this section, we describe a few specific contributions of biocuration to SIB databases in various bioinformatics domains (structured according to the ‘fields of activity’ in Figure 1, although some databases may be assigned to more than one category), and starting with the experience of Swiss-Prot.

### Proteins and Proteomes, systems biology

Through ExPASy, SIB provides a panoply of resources; from the description of individual protein sequences up to whole biological systems, from tools for protein prediction and identification from nucleotide sequences or mass spectrometry to 2D gel electrophoresis data, as well as methods and software for protein classification, functional annotation, similarity search and alignment and finally imaging tools.

**UniProtKB/Swiss-Prot** (www.uniprot.org) provides comprehensive expert-curated functional annotation for over 540 000 protein sequences, including descriptions of protein function, location, interactions and domain structure, as well as post-translational modifications (PTMs) and variants and their functional impact (18). UniProtKB/Swiss-Prot curation makes use of well-developed standards and ontologies such as the Gene Ontology (GO) (19) and **ENZYME** (http://enzyme.expasy.org), a reference for the hierarchical enzymatic classification of the IUBMB/IUPAC joint nomenclature committee that is maintained by SIB. Explicit representations of enzymatic reactions are also provided by **Rhea** (www.ebi.ac.uk/rhea), a comprehensive and non-redundant resource of expert-curated biochemical reactions (20) described using species from the ChEBI (Chemical Entities of Biological Interest) ontology of small molecules (21), for which Rhea is a major source of data. Rhea has been designed for the functional annotation of enzymes in UniProtKB and for the description of genome-scale metabolic networks and models such as those provided by the **MetaNetX** (www.metanetx.org) model repository and analysis platform (22). Rhea (and UniProtKB/Swiss-Prot) are extensively used by **SwissLipids** (www.swisslipids.org), an expert-curated resource for lipid biology developed by SIB and the Swiss initiative for systems biology SystemsX.ch. SwissLipids links mass spectrometry analytical outputs to a library of over 300 000 possible lipid structures and curated information on their metabolism and occurrence (23). An integration and visualization of experimental proteomics evidence onto the predicted topology of membrane proteins and in the context of the wealth of UniProtKB annotations is provided by **Protter**, a recently developed software tool (24).

Accurate expert annotation is an absolute prerequisite for the development of reliable and high-quality computational methods for automated annotation. **UniProtKB/TrEMBL** (unreviewed and automatically annotated) makes extensive use of expert-curated resources from SIB such as **PROSITE** (http://prosite.expasy.org) (25) and **HAMAP** (http://hamap.expasy.org) (26), which are both available as web-based resources for external users to annotate their own protein and proteome sequences.

UniProtKB furthermore provides links to more than 150 collections of biological data, including many of the other expert-curated SIB resources described in more detail below.

**neXtProt** (www.nextprot.org) is an innovative knowledge platform focusing on human proteins (27). Built on top of the UniProtKB/Swiss-Prot annotation corpus, it provides additional expert-curated information on protein expression, subcellular localization, PTMs and protein variations, gathered from selected high-quality, large-scale experimental data sets. It has been chosen as the reference database for the Human Proteome Project (HPP) of the Human Proteome Organization (HUPO), which aims at providing experimental validation for every human protein. As such, it collects and displays all the mass spectrometry data generated by this consortium (28). The neXtProt database can be queried through an innovative search engine allowing to do very complex and precise queries, not only across the annotated data sets, but also taking advantage of external, RDF-based resources. neXtProt's application programming interface allows to quickly build new applications on top of the existing platform. The curation workflow is supported by an original editor (called **BioEditor**), powered with semantic web technologies and by a set of text analytics tools, jointly developed with the SIB Text Mining group. The text mining tools are currently being integrated in order to prioritize research articles for the annotation of both diseases and protein functions (29,30).

**STRING** (www.string-db.org) (4) is another popular SIB database, focusing on known and predicted protein-protein interactions. It is produced and maintained in collaboration with partners at EMBL Heidelberg, University of Copenhagen, and the MPI for Molecular Cell Biology and Genetics in Dresden. It includes direct (physical) as well as functional associations, derived from automated text-mining of the literature, analysis of high-throughput experiments, curated pathway databases, (conserved) co-expression, and *de novo* interaction predictions using genomic context. The current release 10.0 covers interactions in more than 2000 organisms; where applicable, interactions are transferred between these organisms based on a hierarchical orthology framework (31). STRING is a typical ‘meta-resource’, aiming to add value to existing, disparate data—in this case by integrating, benchmarking, scoring and disseminating protein interactions in a user-friendly fashion. It offers further services, such as statistical analysis of a given user input, to uncover enriched functional pathways and network topology trends, as well as network clustering functionality. In contrast to the databases mentioned above, the STRING database is not manually curated, but it does complement SIB's data resources and is in fact among the most widely used SIB resources.

**UniCarbKB** (www.unicarbkb.org) collects comprehensive information on published glycan/carbohydrate structures and published glycoprotein information spanning global and site-specific glycan attachments where available. In the past decades, most structures of glycans have been solved after being cleaved off their natural support (e.g. glycoproteins or glycolipids). Conversely, most protein glycosylation sites have been mapped and stored independently of the sugar structure, e.g (32). As a result, the correlation between glycan structures and glycoprotein sites is most of the time lost in glycomics databases, e.g. (33), and is only implicit in protein databases. The collection of site-specific glycan structural information was initiated by the team of Prof. N. Packer in 1999 and resulted in the release of GlycoSuiteDB in 2001 (34) as a curated database of glycan structures and their protein attachment, where known. In 2011, Prof. Packer joined forces with SIB to launch a wider project of interconnected glycoproteomics resources starting with UniCarbKB (35) that integrates and updates GlycoSuiteDB and links experimental data to the glycan structures. Protein glycosylation site annotation in UniProtKB and neXtProt benefits from the input of the UniCarbKB consortium by reciprocal cross-referencing. To complement the description of protein glycosylation, SIB has developed **SugarBindDB** (http://sugarbind.expasy.org) (36), a manually curated database of interactions between pathogen lectins and carbohydrate ligands of mammalian hosts. This work emphasizes the significance of resource integration. Cross-links between SugarBindDB, UniCarbKB and UniProtKB are shown to provide insight into host-pathogen interactions: starting with a glycan ligand of a pathogen lectin reported in SugarBindDB and mapping it to the glycan structures in UniCarbKB leads to a shortlist of glycoproteins reported to bear the matching glycans. This information then suggests a set of potential pathogen lectin-host glycoprotein interactions available for further investigation.

### Genes and genomes

Several SIB resources focus on the prediction of regulatory sequence motifs in genome sequences, provide information on gene variants (including sequence variations and polymorphisms), assess copy number variation or study gene expression evolution in different organisms. They play important roles in understanding how changes in gene sequence information have a role on the regulation and expression of transcripts, and on the function of protein-coding genes.

**SwissRegulon** (37) is a database of genome-wide annotations of regulatory motifs, promoters and transcription factor binding sites in promoter regions across a collection of model organisms (17 prokaryotes and baker's yeast), and two mammals (mouse, human). It is hosted at the SwissRegulon portal (http://swissregulon.unibas.ch), a repository of databases and bioinformatics tools related to regulatory genomics. SwissRegulon's promoter annotations in mammals are based on the integrated analysis (38) of high-throughput CAGE data from the FANTOM projects (39,40) together with collections of known transcription structures. The regulatory site predictions were obtained using rigorous Bayesian probabilistic methods that operate on orthologous regions from related genomes, and use explicit evolutionary models to assess the evidence of purifying selection on each predicted site (41). All SwissRegulon's data are accessible through an interactive genome browser with search functions, as well as downloadable flat files.

**EPD** (Eukaryotic Promoter Database) (http://epd.vital-it.ch) is an annotated database of eukaryotic POL II promoters for which the transcription start site has been experimentally defined (42). Due to large amounts of newly available transcript mapping data, the number of promoter coverage for mouse has more than doubled in the last year, reaching 21 239 promoters and over 90% gene coverage for the two most important organisms: human and mouse. Today, individual promoter entries are automatically generated from NGS data by carefully designed and extensively tested data processing pipelines. The traditional manual curation efforts applied to each individual entry have been replaced by rigorous quality controls of the input data and by visual sanity checks of randomly sampled entries generated by a data processing pipeline under construction. The latter involves comparison of automatically generated promoter annotations with the underlying primary data to make sure that they are consistent with human interpretation.

The **arrayMap** (www.arraymap.org) (43) database provides over 60 000 pre-processed DNA copy number profiles from human cancer genome studies. This resource, the largest of its kind, facilitates the identification of cancer specific copy number mutation patterns, and facilitates the association of potential target genes with affected tumour entities. The latest release introduced a range of new features, including an HTTP-based data API. This allows users to access directly the group's pre-processed cancer genome data and to use them in their own downstream applications. Data for this repository are curated from probe-specific genomic array data, accessed either through public repositories, e.g. NCBI GEO (44), EBI ArrayExpress (45) or from online supplements of published cancer genome studies. Besides curating annotated oncogenomic data from research articles, the related **Progenetix** (www.progenetix.org) (46) repository also provides comprehensive information about scientific publications reporting original analyses of cancer genomes, including geographic and contact information as well as number of samples analysed. Together, arrayMap and Progenetix facilitate the integration of cancer genome data into pharmacogenomic detection pipelines and clinical diagnostics but also promote collaborative data sharing and analysis projects.

**Bgee** (www.bgee.org) (47), a database for gene expression evolution, has grown from 5 to 17 animal species in the last 12 months, with the notable addition of a wealth of RNA-seq and Affymetrix microarray gene expression data. The workflow used in Bgee allows for the integration and comparison of expression data for any animal species, as distant as, e.g. human and nematode. The recent increase in covered species numbers was a result of a tight collaboration with the GO (19) and other projects on the Uberon anatomical ontology (48), as well as authors’ new methods for the development of anatomical and developmental annotations, for the quality control of source raw expression data and for the assessment of confidence in annotations.

Bgee strives for delivering a curated gene expression data set of the highest quality. For this, the Bgee team has notably contributed to the development of major ontologies such as Uberon, and led the development of new ontologies such as the *developmental stage ontologies* (see https://github.com/obophenotype/developmental-stage-ontologies/). It has also developed new methods, or implemented existing ones, to filter low-quality and redundant transcriptomics data. The Bgee team has developed a new quality control for Affymetrix data, outperforming other methods in identifying poor-quality arrays (49), and has uncovered hidden duplicated content in public transcriptomics data, affecting about 14% of the data annotated by Bgee (50). These principles are now applied to the curation of RNA-Seq data, the Bgee workflow allowing for the filtering of low-quality or redundant RNA-Seq libraries. In addition to the curation of expression data, Bgee also provides annotations of homology relations between anatomical structures (https://github.com/BgeeDB/anatomical-similarity-annotations/), allowing for the automated comparison of the anatomy of any animal species. Because of the intrinsic uncertainty of the evidence sources used for defining homology relations, it has become essential to be able to assess and capture their level of reliability. This work led to the creation of the *Confidence Information Ontology* (51), as well as a proposed workflow for the integration of multiple evidence sources, in collaboration with several groups from the biocuration community, notably the Swiss-Prot and neXtProt groups at SIB. As a result of this work, Bgee is to date one of the only resources capable of integrating and comparing gene expression data in any animal species.

### Structural bioinformatics, drug design and health

SIB has a long-lasting tradition of molecular structure related databases and tools. It began in the early 1990's with **SWISS-MODEL** (5,52)—the first fully automated protein modelling server—and **Swiss-PDBviewer** (http://spdbv.vital-it.ch, also known as DeepView), an application to align, superimpose, analyse and visualize protein structures. Since it was running on standard desktop computers, many people were suddenly able to access the molecular modeling field, and it was extensively used for teaching. The corresponding papers were the highest cited Swiss resource articles for several years (53,54) and paved the way for the development of these popular resources (55).

**SWISS-MODEL** (http://swissmodel.expasy.org) is a widely used automated protein structure homology-modelling server for generating 3D models of protein structures and complexes. It uses information from homologous protein structures (templates) to build models for target protein sequences. SWISS-MODEL relies on the SWISS-MODEL Template Library (SMTL), a curated database of experimental structures, to ensure that accurate and up-to-date structure information is used during the model building process. **SWISS-MODEL Repository** (http://swissmodel.expasy.org/repository) (6) is a database of annotated 3D comparative protein structure models built using the SWISS-MODEL pipeline for amino acid sequences of selected model organisms from the UniProt knowledge base. Mapping annotation and cross-references from resources such as UniProt and STRING allows interpretation of sequence-based annotation in the context of 3D structures. In order to allow querying for all structure information available for a protein, the **Protein Model Portal** (www.proteinmodelportal.org) (56) of the Structural Biology Knowledgebase project (57) federates theoretical models with experimental structures (58) in a single portal. Homology models are used in a broad spectrum of applications in life science research when direct experimental structures are not available. However, the accuracy of a model determines its suitability for specific applications (59). For this reason, new statistical evaluation methods such as **QMEAN** (http://swissmodel.expasy.org/qmean) (60) and **QMEANBrane** (61), have been developed in the past years to estimate the quality of structure models for soluble and transmembrane proteins.

The understanding of 3D molecular structures has its main application in design and development of new drugs. **SwissDrugDesign** is a large collection of resources developed at SIB covering all aspects of computer-aided drug design including the databases **SwissSidechain** (www.swisssidechain.ch) (62,63) and **SwissBioisostere** (www.swissbioisostere.ch) (64). SwissSidechain gathers expert-curated information on hundreds of commercially available non-natural amino acids for *in silico* peptide design. SwissBioisostere collects several million molecular substructural replacements, their frequency of use and performance in biochemical assays extracted from the literature. It was developed using data associated with the highest levels of confidence according to the ChEMBL curators. Indeed, high-quality and trustworthy data on the chemical structure of small molecules and their biological activity is an absolute prerequisite not only for generating reliable structure-activity relationships that open the road to the design of new potent compounds, but also more generally for developing novel efficient computational methods for drug design. Collecting and curating high-quality data from medicinal chemistry journals and patents regarding existing ligands of a therapeutically-relevant protein is therefore an activity that is often performed at early stages of any drug design effort. As an example, the development of **iLOGP** (65), an in-house n-octanol/water partition coefficient (log P) estimator, required collecting more than 17,500 small molecules along with their experimental log P values from several publicly available databases. This wealth of data was subject to an intense expert manual curation process to homogenize the molecular structure format and resolve duplicates, but also to detect and correct missing, ambiguous or erroneous data. iLOGP is available from the **SwissADME** (www.swissadme.ch) web tool, which calculates physicochemical parameters for small molecules in relation with pharmacokinetic, pharmacodynamic and druglikeness properties. When the amount of data is too large for manual curation, automated cleansing can be performed in order to increase the data relevance and quality. *In silico* molecular screening is a typical example, which requires each small molecule to be checked on beforehand for chemical structure inconsistencies, cleaned from counter-ions and other accompanying compounds, checked for the most probable tautomer, neutralized or protonated at a given pH value and potentially transformed into a 3D conformation. This automated curation process was applied meticulously during the development of the reverse screening tool **SwissTargetPrediction** (www.swisstargetprediction.ch) (66–68) and for the future ligand-based screening tool **SwissSimilarity** (www.swisssimilarity.ch).

### Evolution and phylogeny

**OMA** (Orthology MAtrix, www.omabrowser.org) (69) is a resource for identifying orthologs among complete genomes. The public database is updated twice a year. In its 19^th^ release, OMA covers 1970 genomes with 10,129,468 predicted genes from all domains of life. Besides its large scope, the distinctive features of OMA are the high specificity of the inferred orthologs, the availability of data in a wide range of formats and interfaces, and a feature-rich web interface providing access to different orthologous groups, gene function predictions, and a synteny viewer to explore the genomic context of orthologs. With *OMA standalone* we provide an open-source implementation of the OMA algorithm enabling researchers to analyse custom genome data in-house.

**OrthoDB** (www.orthodb.org) (70) is the hierarchical catalog of orthologous protein-coding genes across a wide variety of species ranging from vertebrates, arthropods, fungi, metazoans to bacteria, identifying ‘equivalent’ genes across species. Refining the concept of homology, orthology allows for the most precise inferences of gene functions from model species to the others, and it is the cornerstone for evolutionary comparative studies. OrthoDB provides a worldwide leading coverage, representing 3028 genomes in 2014 and growing to over 4300 genomes in the upcoming release in 2015. Notably, OrthoDB provides the most comprehensive sampling of animal genomes. OrthoDB strives for greater gene coverage while keeping high accuracy of predictions. While simpler to identify, single-copy orthologs can be found by most of the methods, greater sensitivity and correspondingly coverage require resolving of more complex gene relations. OrthoDB software is publicly available (www.orthodb.org/orthodb_software), and it was shown to perform best (69) on a benchmarking set of manually curated orthologs. In addition to the most extensive integration of functional annotations from UniProt, InterPro (71), GO (19), OMIM (72), model organism phenotypes and COG functional categories, OrthoDB uniquely provides evolutionary annotations including rates of ortholog sequence divergence, copy-number profiles, sibling groups and gene architectures. The text searches at the web user interface allow the use of complex logic with various identifiers of genes, proteins, domains, ontologies, and annotation keywords and phrases. In spite of the dramatic data growth, OrthoDB has maintained the option to query the database by online homology searches. Users can select the relevant orthology level by the NCBI taxonomy and specify species of interest to keep the reported results readable. Gene copy-number profiles can also be queried. OrthoDB provides the base data for BUSCO methodology to assessments of genome assembly and annotation completeness (http://buscos.ezlab.org) (73), as well as for CEGA (http://cega.ezlab.org) a catalog of conserved elements from genomic alignments.

## COMPETENCE CENTRES AND INFRASTRUCTURE

Most of SIB's resources are accessible to a large international user community via web interfaces. These resources need to be hosted on dedicated computational infrastructures and supported by professional staff. The SIB **Vital-IT** group (www.vital-it.ch) provides computational infrastructure, development support and bioinformatics expertise to the life science community. SIB also co-manages the Center for Scientific Computing (**sciCORE** http://scicore.unibas.ch), and collaborates with the Service and Support for Science IT (**S3IT** www.s3it.uzh.ch) facility and the Scientific Information Services (**SIS** www.sis.id.ethz.ch). Additionally, local and regional life science users are supported by direct access to high-performance computing infrastructures. Therefore, SIB supports and collaborates with life scientists directly (including through active participation in life science research), and offers services such as analysis of high-throughput data (genome/exome sequencing, RNA sequencing, proteomics), scientific support of (bio)medical projects, development of algorithms, biostatistics training as well as helpdesk and support.

These competence centres are complemented by dedicated bioinformatics core facilities that are mainly specialized in supporting local and regional life scientists: Bioinformatics Core Facility (**BCF** http://bcf.isb-sib.ch), Bioinformatics and Biostatistics Core Facility (**BBCF** http://bbcf.epfl.ch), the Bioinformatics Unravelling Group (**BUGFri** www.unifr.ch/bugfri/), the Interfaculty Bioinformatics Unit (**IBU** www.bioinformatics.unibe.ch) and the bioinformatics facility at **FMI** (www.fmi.ch/research/platforms/platform.html?plt=115).

## TRAINING

The need for bioinformatics training across diverse life science disciplines is high, and is expected to increase significantly with the challenges posed by personalized health and medicine, and big data. Training is an important SIB resource and is conceived to ensure that life and health scientists make the best use of bioinformatics in general, and more specifically of SIB's resources in their research.

The SIB training programme is prepared with care. It takes into account the different levels, needs and backgrounds of the SIB training audience, which is composed of scientists in academia and industry from all over the world. The program also evolves constantly to cover new methodological and technical trends. The 2016 training programme, for instance, is composed of short and long face-to-face courses covering topics such as large-scale data analysis, statistics, algorithms and methods for bioinformatics, network analysis, basic computational tools and the use of HPC in biological research. It also includes courses on specific SIB resources, some of which have been described above. These courses are organized by domains, covering for example the protein resources (with UniProtKB/Swiss-Prot, neXtProt, STRING), the genomic resources (with EPD and other SIB tools) and structural biology (with SWISS-MODEL, Swiss-PdbViewer and SwissDrugDesign).

## CLINICAL BIOINFORMATICS

One of the emerging application domains of SIB knowledge bases, tools and infrastructure is in the area of health for which professional services, reliable infrastructure and expertise is required.

SIB has a long presence in the biomedical world, providing services such as neXtProt or arrayMap. In the recent past, activities in this area intensified with the creation, among others, of tools for rapid and reliable analyses of non-invasive prenatal NGS testing of aneuploidies on fetal cfDNA circulating in maternal blood (Prendia: www.prendia.ch/en/) (74). Anticipating the need to develop clinically-useful bioinformatics tools and warehouses to integrate, analyse and interpret the emerging flood of molecular data soon available to clinicians, in 2012 SIB decided to create a *clinical bioinformatics unit*, whose scope covers all omics areas as well as other high-throughput data. Its mandate is to come forward with harmonized, interoperable solutions that take into account the clinical daily needs as expressed by those involved in healthcare, taking advantage of the added value provided by the expertise and knowledge present within the various SIB groups.

## CONCLUDING REMARKS

One of SIB's major aims is to provide sustainable data resources to the life sciences community. This has been done consistently and meticulously for the best part of three decades—indeed, a few of the resources predate the official creation of SIB in 1998. This is particularly true for the knowledge bases, tools and infrastructure that are the fruit of dedicated people who exert rigorous curational efforts and coordination. Bioinformatics and particularly data science have a major impact not only on science but also on our daily lives; data interpretation and curation are of key importance for SIB and its experts. Modelled on Switzerland's federal structure, the SIB is organized as a federation of bioinformatics research and service groups. This unique representation has set precedence in organizing bioinformatics nationally, and its example is now being followed by many other European countries. Previous and current SIB activities demonstrate SIB's outstanding role in bioinformatics in Europe (incl. ELIXIR www.elixir-europe.org) and across the world.

## APPENDIX

**SIB Swiss Institute of Bioinformatics’ Members**

The following SIB members (a *subset* of all current SIB members) are **co-authors** of this article since they participated directly or indirectly in at least one of the resources and/or Swiss bioinformatics activities mentioned in this article.

- An up-to-date list of *all* SIB members can be found on SIB's web site (www.isb-sib.ch) under ‘**Finding People**’.
- Scientific contact for resources: helpdesk@expasy.org or www.expasy.org/support.

Lisandra Aguilar Bultet, José Aguilar-Rodríguez, Christian H. Ahrens, Erik Lennart Ahrné, Ni Ai, Lucila Aimo, Altuna Akalin, Tyanko Aleksiev, Davide Alocci, Adrian Altenhoff, Isabel Alves, Giovanna Ambrosini, Pascale Anderle Pedone, Paolo Angelino, Maria Anisimova, Ron Appel, Ghislaine Argoud-Puy, Konstantin Arnold, Bulak Arpat, Panu Artimo, Kelly Ascencao, Andrea Auchincloss, Kristian Axelsen, Vivienne Baillie Gerritsen, Amos Bairoch, Parit Bansal, Delphine Baratin, Alessandro Barbato, Valérie Barbié, David Barras, Maria Barreiro, Sophie Barret, Frederic Bastian, Teresa Manuela Batista Neto, Michael Baudis, Emmanuel Beaudoing, Jacques S Beckmann, Amel Kawter Bekkar, Leila Ben Hamida Cammoun, Sara Benmohammed, Madeleine Bernard, Claire Bertelli, Martino Bertoni, Stefan Bienert, Olivier Bignucolo, Aivett Bilbao, Adem Bilican, Diana Blank, Marie-Claude Blatter, Lorenz Blum, Jocelyne Bocquet, Brigitte Boeckmann, Jerven Tjalling Bolleman, Lorenza Bordoli, Lars Bosshard, Gerard Bouchet, Lydie Bougueleret, Emmanuel Boutet, Christophe Bovigny, Sinisa Bratulic, Lionel Breuza, Alan James Bridge, Aurore Britan, Francisco Brito, Josias Brito Frazão, Rémy Bruggmann, Philipp Bucher, Frédéric Burdet, Lukas Burger, Elena Maria Cabello, Ruben Martin Cabezon Gomez, Sandra Calderon, Gina Cannarozzi, Sarah Carl, Cristina Casals Casas, Sebastien Catherinet, Rouayda Cavin Périer, Christophe Charpilloz, Prasad Datatray Chaskar, Weihua Chen, Anush Chiappino Pepe, Bastien Chopard, Hoi Yee Chu, Natacha Civic, Manfred Claassen, Sylvie Clottu, Martino Colombo, Isabelle Cosandier, Elisabeth Coudert, Isaac Crespo, Marc Creus, Béatrice Cuche, Michel A. Cuendet, Isabelle Cusin, Neha Daga, Antoine Daina, Jérôme Dauvillier, Fabrice David, Iakov Davydov, Mariana De Sa Ricca Manadelo Ferreira, Tjaart de Beer, Edouard de Castro, Charles de Santana, Julien Delafontaine, Mauro Delorenzi, Céline Delucinge-Vivier, Ömer Demirel, Robert Derham, Emmanouil Manolis Dermitzakis, Linda Dib, Seydina Diene, Nahzli Dilek, Julian Dilmi, Marcin Jakub Domagalski, Julien Dorier, Dolnide Dornevil, Aline Dousse, René Dreos, Pablo Duchen, Paula Duek Roggli, Isabelle Dupanloup Duperret, Christine Durinx, Séverine Duvaud, Robin Engler, Serap Erkek, Pablo Escobar López, Anne Estreicher, Laurent Excoffier, Roberto Fabbretti, Jean-Luc Falcone, Laurent Falquet, Maria Livia Famiglietti, Anne-Maud Ferreira, Marc Feuermann, Marc Filliettaz, Volker Flegel, Adrien Foucal, Andrea Franceschini, Geoffrey Fucile, Dimos Gaidatzis, Victor Garcia, Elisabeth Gasteiger, Alain Gateau, Lorenzo Gatti, Pascale Gaudet, Arnaud Gaudinat, Sebastien Gehant, David Gfeller, Walid H. Gharib, Marie Ghraichy, Cindy Gidoin, Manuel Gil, Anne Gleizes, Julien Gobeill, Gaston Gonnet, Arnaud Gos, Lou Gotz, Alexandre Gouy, Djordje Grbic, Romain Groux, Nadine Gruaz-Gumowski, Delphine Grun, Andreas Gschwind, Nicolas Guex, Saumya Gupta, Michael Gétaz, Dennis Haake, Juergen Haas, Vassily Hatzimanikatis, Gerald Heckel, Daniel Federico Hernandez Gardiol, Valérie Hinard, Ursula Hinz, Krisztian Homicsko, Oliver Horlacher, Sayed-Rzgar Hosseini, Hans-Rudolf Hotz, Chantal Hulo, Christian Hundsrucker, Mark Ibberson, Sten Ilmjärv, Vassilios Ioannidis, Panagiotis Ioannidis, Christian Iseli, Robert Ivanek, Justyna Iwaszkiewicz, Philippe Jacquet, Martin Jacquot, Vidhya Jagannathan, Maxime Jan, Jeffrey Jensen, Maria U. Johansson, Niklaus Johner, Florence Jungo, Thomas Junier, Abdullah Kahraman, Maria Katsantoni, Guillaume Keller, Arnaud Kerhornou, Fahad Khalid, Dirk Klingbiel, Andrea Komljenovic, Evgenia Kriventseva, Nadezda Kryuchkova, Sunil Kumar, Zoltan Kutalik, Dmitry Kuznetsov, Rostyslav Kuzyakiv, Lydie Lane, Vicente Lara, Leonardo Ledesma, Marion Leleu, Philippe Lemercier, Daniel Lew, Damien Lieberherr, Robin Liechti, Frederique Lisacek, Heidi Lischer, Glenn Litsios, Jialin Liu, Thierry Lombardot, Aurélien Macé, Sergio Maffioletti, Mohamed-Ali Mahi, Massimo Maiolo, Somi Reddy Majjigapu, Lars Malmström, Véronique Mangold, Diana Marek, Julien Mariethoz, Ray Marin, Olivier Martin, Xavier Martin, Trinidad Martín-Campos, Camille Mary, Frédéric Masclaux, Patrick Masson, Cécile Meier, Antonio Messina, Muriel Metrailler Lenoir, Xavier Meyer, Pierre-André Michel, Olivier Michielin, Alessio Milanese, Edoardo Missiaglia, Jorge Molina Perez, Vanessa Monteiro Caria, Philippe Moret, Sebastien Moretti, Anne Morgat, Anaïs Mottaz, Luc Mottin, Yoann Mouscaz, Markus Mueller, Riccardo Murri, Roman Mylonas, Samuel Neuenschwander, Frederic Nikitin, Anne Niknejad, Nevila Nouspikel, Lydie Nso Nso, Michal Okoniewski, Ulrich Omasits, Benjamin Paccaud, Mikhail Pachkov, Salvo Giacomo Paesano, Marco Pagni, Patricia M. Palagi, Emilie Pasche, Joshua L. Payne, Ivo Pedruzzi, Stephan Peischl, Manuel Peitsch, Sabine Perlini, Sandrine Pilbout, Michael Podvinec, Rainer Pohlmann, Davide Polizzi, Douglas Potter, Sylvain Poux, Monica Pozzato, Sylvain Pradervand, Viviane Praz, Manuela Pruess, Eva Pujadas, Julien Racle, Marcelo Raschi, Osman Ratib, Antonio Rausell, Valentine Rech de Laval, Nicole Redaschi, Christine Rempfer, Guangpeng Ren, Reza Ali Rezaee Vahdati, Leonor Rib, Oksana Riba Grognuz, Emma Ricart Altimiras, Catherine Rivoire, Thibault Robin, Marc Robinson-Rechavi, Joao Rodrigues, Bernd Roechert, Patrick Roelli, Valentina Romano, Gregoire Rossier, Alexander Roth, Jacques Rougemont, Julien Roux, Hélène Royo, Patrick Ruch, Michela Ruinelli, Mohamad Rustom Abdul Sater, Ute F. Röhrig, Sina Rüeger, Nicolas Salamin, Martial Sankar, Namrata Sarkar, Moritz Saxenhofer, Mathieu Schaeffer, Yolanda Schaerli, Elke Schaper, Annette Schmid, Emanuel Schmid, Christoph Schmid, Michael Schmid, Sebastian Schmidt, Daniel Schmocker, Michel Schneider, Thierry Schuepbach, Torsten Schwede, Frédéric Schütz, Thierry Sengstag, Martha Serrano, Atul Sethi, Omid Shahmirzadi, Christian Sigrist, Daniele Silvestro, Felipe Aristides Simao Neto, Cedric Simillion, Milan Simonovic, Nives Skunca, Kasia Sluzek, Charlotte Soneson, Kathleen Sprouffske, Michael Stadler, Sylvie Staehli, Brian Stevenson, Heinz Stockinger, Jakub Straszewski, Thomas Stricker, Gabriel Studer, Andre Stutz, Madeleine Suffiotti, Shyamala Sundaram, Damian Szklarczyk, Peter Szövenyi, Fredrik Tegenfeldt, Daniel Teixeira, Susanne Tellenbach, Adam Alexander Thil Smith, Michael Tognolli, Ivan Topolsky, Thuong Van Du Tran, Petros Tsantoulis, Athanasia C. Tzika, Asier Ullate Agote, Erik van Nimwegen, Christian von Mering, Adithi Varadarajan, Maren Veranneman, Laure Verbregue, Anne-Lise Veuthey, Dina Vishnyakova, Rounak Vyas, Andreas Wagner, Daniel Walther, Hon Wai Wan, Mingcong Wang, Robert Waterhouse, Andrew Waterhouse, Adrian Wicki, Leonore Wigger, Pratyaksha Wirapati, Ursula Witschi, Stefan Wyder, Kurt Wyler, Daniel Wüthrich, Ioannis Xenarios, Kana Yamada, Zheng Yan, Haleh Yasrebi, Monique Zahn, Nadine Zangger, Evgeny Zdobnov, Daniel Zerzion, Vincent Zoete, Stefan Zoller.

**Acknowledgements to Additional SIB Members (non-co-authors)**

The authors would like to explicitly thank the following SIB members not mentioned in the co-author list above:

Luciano Abriata, Thierry Aebischer, Murodzhon Akhmedov, Antonio Alves Meireles Filho, Stefano Andreozzi, Naara Appel, Florence Armand, Alberto Arribas, Samira Asgari, Meric Ataman, Martina Audagnotto, Deniz Aydin, Pierre Bady, Claudia Bank, Diana Barac, Benoîte Bargeton, Joelle Barido-Sottani, Caterina Barillari, Istvan Bartha, Angela Bean, Johannes Becker, Niko Beerenwinkel, Gwendoline Bellement, Fabrizio Benedetti, Sven Bergmann, Simon Berneche, Roel Bevers, Daniel Biasse, Manuel Bichsel, Jonathan Bieler, Ana Paula Bittencourt Piccini, Marcelo Boareto do Amaral, Christian Bolliger, Veronika Boskova, Andrew Brown, Julien Bryois, Alfonso Buil, Rosamaria Cannavo, Mathias Cardner, Francesco Nicola Carelli, Santiago Carmona, Luciano Cascione, Anirikh Chakrabarti, Nimisha Chaturvedi, Sarvenaz Choobdar, Claire Clivaz, Simona Constantinescu, Tanguy Corre, Diego Claudio Cortez Quezada, Roberto Croce, Anne Cuendet, Jing Cui, Riccardo Dainese, Matteo Dal Peraro, Tiziano Dallavilla, Luis De Oliveira, Nicolas Delaleu, Olivier Delaneau, Bart Deplancke, Lekshmi Dharmarajan, Madeline Diekmann, Christos Dimitrakopoulos, Beatrice Dimitriades-Schmutz, Yoana Aleksandrova Dimitrova, Simon Dirmeier, Maria Pamela Dobay, Michaël Dougoud, Julien Duc, Guillaume Dumont, Louis du Plessis, Daniel Dörr, Kristina Elfstroem, Franz-Josef Elmer, Gregory Bruce Ewing, Imran Fanaswala, Jacques Fellay, Chrystel Feller, Georgios Fengos, Anna Ferrer, Sylvie Fiaux, Chris Field, Iris Finci, Anamarija Fofonjka, Giulia Fonti, Patrick Fried, Michael Frochaux, Juan Fuentes, Luca Galbusera, Marco Galimberti, Manuel Garcia-Albornoz, Ramon Garcia-Escudero, Marco Garieri, Lada Georgieva, Cristina Gabriela Ghiurcuta, Souvik Ghosh, Alexandra Gnann, Robert Gnuegge, Monica Golumbeanu, Harold Gomez, Jérôme Goudet, Andreas Johannes Gruber, Franziska Gruhl, Sarah Guethe, Elsa Guillot, João Guimarães, Rafal Woiciech Gumienny, Rudiyanto Gunawan, Kyle Gustafson, Foivos Gypas, Noushin Hadadi, Tuure Hameri, Christian Hammer, Florian Heer, Davide Martin Heller, Micha Hersch, Ariane Leoni Hofmann, Cédric Howald, Sandro Hutter, Dagmar Iber, Kristen Irwin, Katharina Jahn, Dominik Jedlinski, Vinay Jethava, Hadi Jorjani, Thomas Julou, Henrik Kaessmann, Alexandra Kalantzi, Alexander Kanitz, Adamandia Kapopoulou, Eleni Karamasioti, Zahra Karimaddini, Irene Keller, Alexandros Kiparissides, Manuel Kohler, Athanasios Kousathanas, Jack Kuipers, Piotr Kupczyk, Ivo Kwee, Denise Kühnert, Lakshmi Narayanan Lakshmanan, David Lamparter, Brian Lang, Sacha Laurent, Stefan Laurent, Deborah Leigh, Yu Lin, Helen Lindsay, Vivian Link, Janina Linnik, Thomas Liphardt, Yang Liu, Alexis Loetscher, Antti Olavi Luomi, Kirill Lykov, Dania Machlab, Carsten Magnus, Anne Maillard-Wermelinger, Alexander Malafeev, Anna-Sapfo Malaspinas, Roger Mallol Parera, Erica Manesso, Liana Manukyan, Sarah Natalia Mapelli, Daniel Marbach, Maria Josefina Marcaida Lopez, Marisa Marciano Wynn, Georges Martin, António F. Martins, Katarina Matthes, Christian Mazza, Aaron McDaid, Jérôme Mermet, Odyssé Michos, Michel C. Milinkovitch, Aristotelis Misios, Ljubisa Miskovic, Venelin Mitov, Abhishek Mitra, Nitish Mittal, Hesam Montazeri, Bernard Moret, Felix Naef, Nishanth Nair, Damien Lionel Nicolas, Stefan Nicolet, Heeju Noh, Malgorzata Nowicka, Ove Oeyaas, Saeed Omidi, Halit Ongen, Louise Ormond, Diana Ottoz, Nikolaos Panousis, Nan Papili Gao, Hassan Pezeshgi Modarres, Jūlija Pečerska, Joana Pinto Vieira, Igor Pivkin, Evarist Planet, Susana Posada Céspedes, Rachana Pradhan, Julien Prados, Dusan Racko, Marie-France Radigois, David Rasmussen, Andrea Riba, Bernd Rinn, Marion Rivoal, Mark Robinson, Simona Rossi, Fabian Rudolf, Rico Rueedi, Hans-Joachim Ruscheweyh, Mikolaj Rybinski, Andrzej Jerzy Rzepiela, Thomas Sakoparnig, Maria Magdalena San Román Rincón, Georgios Savoglidis, Petar Scepanovic, Olivier Schaad, Romy Schleiss, Fabian Schmich, Emanuel Schmid, Ralf Schmidt, Sara Schulthess, Petra Catalina Schwalie, David Seifert, Marianne Seijo, Mingfu Shao, Hyunjin Shim, Jochen Singer, Jonathan Sobel, Keng Cher Soh, Reyhan Sonmez Flitman, Suzanne Soto, Vitor Sousa, Tanja Stadler, Andrzej Stasiak, Ancilla Stefani, Yvonne Steger, Joerg Stelling, Anna Stopka, Isabelle Stévant, Mikael Sunnaker, Afzal Pasha Syed, Giorgio Tamo, Simon Tanaka, Grégory Theiler, Christian Axel Wandall Thorball, Thomas Thurnherr, Monica Roxana Ticlla Ccenhua, Onur Tidin, Milenko Tokic, Macarena Toll Riera, Matteo Tomasini, Stepan Tymoshenko, Minhaz Ud-Dean, Arantxa Urchueguia, Nadia Vertti Quintero, Laura Vinckenbosch, Ana Viñuela, Jannik Vollmer, Sonja Voordijk, Jingkui Wang, Zhongyi Wang, Maria Warnefors, Lukas M. Weber, Daniel Wegmann, Ellis Whitehead, Alexandre Wicky, Lukas Widmer, Min Ye, Florence Yerly, Jake Yeung, Pencho Yordanov, Mihaela Zavolan, Tobias Zehnder, Chi Zhang, Xiaobei Zhou, Aikaterini Zisaki, Benjamin Zoller, Alix Zollinger.
